# Supplementary material for: A practical comparison of the next-generation sequencing platform and assemblers using yeast genome
Source: Life Sci Alliance. 2023 Feb 6;6(4):e202201744. doi: 10.26508/lsa.202201744 (PMC9902641; doi:10.26508/lsa.202201744)
Supplement: Supplementary file 1 [file LSA-2022-01744_TableS1.docx]

**Table S1. The read numbers and total bases of the SGS dataset which were used with GenomeScope.**

|  | **Illumina NovaSeq 6000** | | **MGI DNBSEQ-T7** | |
| --- | --- | --- | --- | --- |
|  | Total read (count) | Total base (Gbp) | Total read (count) | Total base (Gbp) |
| Raw data | 7,090,758 ×2 | 2.14 | 5,012,291 ×2 | 1.5 |
| Trim Galore! (Q30) | 6,925,924 ×2 | 2.08 | 4,340,400 ×2 | 1.29 |
| Trimming ratio | 2.32 % | 2.80 % | 13.40 % | 14 % |
