# Supplementary material for: A practical comparison of the next-generation sequencing platform and assemblers using yeast genome
Source: Life Sci Alliance. 2023 Feb 6;6(4):e202201744. doi: 10.26508/lsa.202201744 (PMC9902641; doi:10.26508/lsa.202201744)
Supplement: Supplementary file 2 [file LSA-2022-01744_TableS2.docx]

**Table S2. GenomeScope results at five distinct *k*-mer levels.**

| **Illumina NovaSeq 6000** | *k* = 15 | *k* = 17 | *k* = 19 | *k* = 21 | *k* = 23 |
| --- | --- | --- | --- | --- | --- |
| Heterozygosity (%) | 0.00488138 | 0.00463503 | 0.00439448 | 0.00412742 | 0.00393381 |
| Genome Haploid Length (bp) | 12,526,885 | 12,567,718 | 12,588,634 | 12,604,522 | 12,618,618 |
| Genome Repeat Length (bp) | 1,583,504 | 625,846 | 520,635 | 502,051 | 494,509 |
| Genome Unique Length (bp) | 10,943,381 | 11,941,872 | 12,067,999 | 12,102,471 | 12,124,109 |
| Model Fit (%) | 99.3186 | 99.2641 | 99.2446 | 99.2549 | 99.2268 |
| Read Error Rate (%) | 0.0880313 | 0.0926146 | 0.0910203 | 0.088977 | 0.0870341 |
|  |  |  |  |  |  |
| **MGI DNBSEQ-T7** | *k* = 15 | *k* = 17 | *k* = 19 | *k* = 21 | *k* = 23 |
| Heterozygosity (%) | 0.134476 | 0.128256 | 0.116555 | 0.106083 | 0.0972866 |
| Genome Haploid Length (bp) | 12,419,501 | 12,432,577 | 12,450,172 | 12,467,010 | 12,480,768 |
| Genome Repeat Length (bp) | 1,585,062 | 617,718 | 514,203 | 498,747 | 492,402 |
| Genome Unique Length (bp) | 10,834,439 | 11,814,859 | 11,935,969 | 11,968,264 | 11,988,366 |
| Model Fit (%) | 94.2486 | 94.1044 | 94.1515 | 94.2828 | 94.3811 |
| Read Error Rate (%) | 0.20375 | 0.208741 | 0.201563 | 0.194053 | 0.187268 |
