# Supplementary material for: A practical comparison of the next-generation sequencing platform and assemblers using yeast genome
Source: Life Sci Alliance. 2023 Feb 6;6(4):e202201744. doi: 10.26508/lsa.202201744 (PMC9902641; doi:10.26508/lsa.202201744)
Supplement: Supplementary file 3 [file LSA-2022-01744_TableS3.docx]

**Table S3. Metrics of subsampled TGS reads.**

| **PacBio** | Total read | Total base (bp) | Longest read (bp) | Mean read length (bp) | N50 (bp) | Mean per read GC contents (%) |
| --- | --- | --- | --- | --- | --- | --- |
| Raw data | 1,784,342 | 16,456,036,400 | 114,447 | 9,222.47 | 13,114 | 35.676 |
| 20× | 27,158 | 250,603,226 | 71,374 | 9,227.60 | 13,179 | 35.673 |
| 30× | 40,741 | 375,900,922 | 63,855 | 9,226.60 | 13,101 | 35.648 |
| 40× | 54,333 | 501,224,025 | 102,191 | 9,225.04 | 13,126 | 35.687 |
| 50× | 67,740 | 626,502,664 | 86,074 | 9,248.64 | 13,157 | 35.668 |
| 60× | 81,453 | 751,802,401 | 83,986 | 9,229.89 | 13,067 | 35.669 |
| 70× | 95,245 | 877,100,948 | 92,757 | 9,208.89 | 13,061 | 35.66 |

| **ONT** | Total read | Total base (bp) | Longest read (bp) | Mean read length (bp) | N50 (bp) | Mean per read GC contents (%) |
| --- | --- | --- | --- | --- | --- | --- |
| Raw data | 283,439 | 4,318,402,163 | 237,199 | 15,235.74 | 32,488 | 34.741 |
| 20× | 16,433 | 250,642,603 | 159,877 | 15,252.40 | 32,759 | 34.744 |
| 30× | 24,424 | 375,919,028 | 180,835 | 15,391.38 | 32,700 | 34.706 |
| 40× | 32,795 | 501,202,426 | 237,199 | 15,282.89 | 32,652 | 34.757 |
| 50× | 41,142 | 626,542,562 | 237,199 | 15,228.78 | 32,750 | 34.728 |
| 60× | 49,089 | 751,832,181 | 186,649 | 15,315.70 | 32,829 | 34.74 |
| 70× | 57,287 | 877,110,338 | 210,445 | 15,310.81 | 32,551 | 34.757 |
