# Supplementary material for: A practical comparison of the next-generation sequencing platform and assemblers using yeast genome
Source: Life Sci Alliance. 2023 Feb 6;6(4):e202201744. doi: 10.26508/lsa.202201744 (PMC9902641; doi:10.26508/lsa.202201744)
Supplement: Supplementary file 4 [file LSA-2022-01744_TableS4.docx]

**Table S4. Homomer counts of subsampled TGS reads.**

| **PacBio (Total base)** | AAAAA | TTTTT | GGGGG | CCCCC | Total |
| --- | --- | --- | --- | --- | --- |
| 20× (250,603,226) | 0.63 % (1,572,899) | 0.73 % (1,824,368) | 0.15 % (363,595) | 0.11 % (281,830) | 1.61 % (4,042,692) |
| 30× (375,900,922) | 0.61 % (2,295,603) | 0.74 % (2,779,917) | 0.14 % (526,178) | 0.11 % (403,628) | 1.6 % (6,005,326) |
| 40× (501,224,025) | 0.62 % (3,121,680) | 0.74 % (3,730,868) | 0.15 % (737,387) | 0.12 % (603,053) | 1.63 % (8,192,988) |
| 50× (626,502,664) | 0.6 % (3,775,351) | 0.75 % (4,673,740) | 0.14 % (874,478) | 0.12 % (778,564) | 1.61 % (10,102,133) |
| 60× (751,802,401) | 0.6 % (4,546,907) | 0.74 % (5,558,631) | 0.14 % (1,078,909) | 0.12 % (933,860) | 1.61 % (12,118,307) |
| 70× (877,100,948) | 0.62 % (5,477,310) | 0.75 % (6,542,327) | 0.15 % (1,273,097) | 0.12 % (1,066,568) | 1.64 % (14,359,302) |

| **ONT (Total base)** | AAAAA | TTTTT | GGGGG | CCCCC | Total |
| --- | --- | --- | --- | --- | --- |
| 20× (250,642,603) | 0.32 % (806,830) | 0.46 % (1,143,975) | 0.03 % (82,703) | 0.01 % (36,116) | 0.82 % (2,069,624) |
| 30× (375,919,028) | 0.32 % (1,201,805) | 0.47 % (1,748,135) | 0.04 % (141,035) | 0.01 % (54,176) | 0.84 % (3,145,151) |
| 40× (501,202,426) | 0.32 % (1,610,027) | 0.46 % (2,285,108) | 0.04 % (206,603) | 0.01 % (71,290) | 0.83 % (4,173,028) |
| 50× (626,542,562) | 0.32 % (2,011,916) | 0.46 % (2,900,619) | 0.04 % (219,374) | 0.01 % (89,890) | 0.83 % (5,221,799) |
| 60× (751,832,181) | 0.32 % (2,420,601) | 0.47 % (3,498,061) | 0.04 % (297,086) | 0.01 % (107,756) | 0.84 % (6,323,504) |
| 70× (877,110,338) | 0.32 % (2,827,919) | 0.47 % (4,080,132) | 0.04 % (343,342) | 0.01 % (125,261) | 0.84 % (7,376,654) |
